# Harmful Algae Impacting Aquatic Organisms: Recent Field and Laboratory Observations

**DOI:** 10.3390/toxins15050339

**Published:** 2023-05-15

**Authors:** Juan José Dorantes-Aranda

**Affiliations:** Independent Researcher, Perth, WA 6000, Australia; juanjodorantes@gmail.com

Algal blooms formed by some phytoplankton species can produce toxins or alter environmental conditions that can affect aquatic organisms and water quality, with impacts on the aquaculture and fisheries industries that can pose a risk to public health. These harmful algal blooms (HAB) appear to be expanding and intensifying in recent decades [1], and their monitoring and management require integrated cooperation amongst scientists, government regulators, seafood industries, health departments, and the public, particularly those that practise recreational fishing and harvesting [2,3]. In the last two decades, the world has witnessed two major harmful algal bloom events, formed by ichthyotoxic species, with catastrophic effects that impacted both wild and farmed organisms. The Arabian Gulf and the Gulf of Oman were impacted by the dinoflagellate *Margalefidinium polykrikoides* (also known as *Cochlodinium polykrikoides*), which caused mass mortalities of finfish, marine mammals, and corals, the closure of desalination plants, and affected tourism activities during 2008–2009 [4]. Blooms by the dictyochophyte *Pseudochattonella verruculosa* affected salmon farming industries in Chile during 2016, causing fish kills with estimated losses of USD800 million [5]. Scientific advances have revolutionised the monitoring and detection of harmful algal species and toxins, including the adoption of analytical methods as alternatives to the mouse bioassay, the use of rapid immunological tests, molecular methods, and biosensors, as well as the development of *in vitro* assays using fish and mammalian cells [6,7,8,9,10,11,12,13]. This Special Issue presents a variety of original studies that show new geographical distribution findings of harmful and toxin-producing species, the adverse effects of toxic species using *in vitro* and *in vivo* assays, a review case study of the current HAB situation in New Zealand, and an analysis of the risks they pose to their aquaculture industry.

The toxic dinoflagellate *Pyrodinium bahamense* was observed in the Mexican coast of the SE Gulf of Mexico for the first time by Núñez-Vázquez et al. Its presence occurred in association with eutrophicated waters near rainwater run-off and wastewater discharge sites. Its ability to produce paralytic shellfish toxins (PST) was confirmed by official analytical methods and rapid tests. Given its potential to form blooms and the occurrence of bivalve harvesting in the area, it was recommended to continue with the monitoring of this and other harmful species to protect public health [14]. The dinoflagellate *Gymnodinium catenatum* is another producer of PST, which impacts aquatic organisms and aquaculture industries in the Gulf of California and the Mexican Pacific. Hernández-Sandoval et al. studied the growth of four strains of this dinoflagellate under variable nitrogen concentrations, which did not alter its toxin profiles but did affect cell growth with a maximum rate of 0.34 day^−1^ at 232 µM (as NaNO_3_) and reaching densities as high as 4000 cells mL^−1^ [15]. The ichthyotoxic dinoflagellate *Margalefidinium polykrikoides* has also affected other parts of the world, including Japan, Korea, China, the United States, and Mexico. Hu et al. successfully employed qPCR technologies in field samples to identify and quantify *M. polykrikoides* along the Chinese coastline, observing maximum concentrations of 100 cells mL^−1^ in the Yangtze River estuary and finding this species on the coast of Tianjin for the first time. Hu et al. recommended the use of this molecular technique as a monitoring and early warning tool for *M. polykrikoides* due to its accuracy and sensitivity [16]. The raphidophyte *Heterosigma akashiwo* is also an ichthyotoxic species that has impacted fisheries in Canada, the United States, Japan, New Zealand, and Chile. Flores-Leñero et al. reported that, under laboratory conditions, the maximum growth of a Chilean *H. akashiwo* strain occurred at 17 °C, salinity of 35 (0.48 day^−1^), with the highest cell densities observed at 12 °C, salinity of 30 (50,000 cells mL^−1^). High concentrations of potentially harmful palmitic, eicosapentaenoic, and stearidonic acids were found in this algal strain, but superoxide radical production was low, and therefore synergistic toxic effects between fatty acids and superoxide radicals were unlikely to occur. Only high algal cell concentrations (47,000 cells mL^−1^) showed significant toxicity towards RTgill-W1 fish gill cells (39% cell viability loss), suggesting a toxic mechanism other than oxidative damage caused by superoxide radicals co-occurring with fatty acids [17]. *Heterosigma akashiwo,* together with *Pseudochattonella verruculosa* and *Alexandrium catenella*, are the main threats to salmon farms in Chile, and international experts have concluded that the RTgill-W1 fish cell assay [12] is currently the best method to assess ichthyotoxicity [5,17,18]. *In vitro* techniques were also employed by Moreira et al. to determine the toxicity of the parasite dinoflagellate *Amyloodinium ocellatum*, which appears to create anoxic and osmoregulatory disturbances in fish followed by bacterial infections. Branchial arch (ABSa15) and caudal fin (CFSa1) cells as well as gilthead seabream erythrocytes were challenged against extracts from *A. ocellatum*, observing strong adverse effects from low-polar extracts obtained from parasitic (trophont) and infective (dinospores) life stages of the dinoflagellate. The ABSa15 cell line and fish erythrocytes were the most sensitive to these extracts (up to 74.5% loss of viability and 100% haemolysis, respectively), confirming the haemolytic and cytotoxic effects of this dinoflagellate [19].

The *in vitro* bioassays discussed so far involve the use of fish cells or erythrocytes, and more assays are being developed to facilitate their performance and ease of use. Allaf and Trick developed a yeast cell assay as an approach to determining toxicity by fish-killing flagellates, using the raphidophyte *Heterosigma akashiwo* and the haptophyte *Prymnesium parvum*, which appear to have similar toxic mechanisms. Yeast cells proved to be sensitive to the two algal species, yeast cell mortalities were observed after 1 h of exposure, and mortality increased with increasing exposure time. Yeast mortality particularly increased when the microalgae were subjected to sonication cycles to rupture the algal cells prior to exposure (37% versus 62% mortality at 1 h and 3 h, respectively, when using *H. akashiwo*), which suggested the release of toxic compounds upon algal cell rupture [20]. Some researchers still use traditional *in vivo* assays employing whole animals; they unequivocally demonstrate adverse effects in an integrated manner as the kinetics of toxins or harmful species tend to play a direct role in the adsorption, distribution, metabolism, and elimination that account for the overall level of toxicity and potential mortality of the whole testing organism [21]. However, fish *in vitro*–*in vivo* extrapolation models are being studied for some environmental toxicants [22]. *In vitro* approaches are gaining popularity in the fields of phycotoxins and food safety [23,24,25] as they overcome societal concerns about the use of animals in experimentation and offer outstanding advantages such as higher reproducibility, sample size, better control, no-stress effect, and ethics approvals [26,27]. However, a correlation between *in vivo* and *in vitro* experimental results is still needed for a conclusive demonstration of the reliability of cell lines as alternatives in toxicology, particularly when assessing ichthyotoxic microalgae [5,28]. Therefore, this is an open invitation to fellow scientists to carry out simultaneous experiments for a direct correlation of *in vitro*–*in vivo* studies considering the now commonly used RTgill-W1 cell line to assess toxicity by harmful microalgae.

The brevetoxin-producing dinoflagellate *Karenia brevis* commonly affects the NE of the Gulf of Mexico, causing mass kills of fish, seabirds, and marine mammals [29]. Litaker et al. investigated the acute survival of red porgy fish larvae, *Pagrus pagrus,* upon exposure to *K. brevis*, observing that larvae survival was inversely proportional to brevetoxin production and directly proportional to the exposure time (mortality at 48 h > 24 h). The effective concentration to kill 50% of the larvae (EC_50_) was estimated at 163 cells mL^−1^ (24 h exposure); this concentration is equivalent to moderately dense *K. brevis* blooms, suggesting that moderate to dense blooms by *K. brevis* may impact fish stocks of ecological and commercial relevance in the Gulf of Mexico [30]. A previous study confirmed the sensitivity of the RTgill-W1 cells towards the two brevetoxins PbTx-2 and PbTx-3 purified from *K. brevis*, the karlotoxin KmTx-2 from the dinoflagellate *Karlodinium veneficum*, and paralytic shellfish toxins from *Alexandrium* spp. [28,31], confirming the suitability of this assay for *in vitro*–*in vivo* correlations. Pease et al. demonstrated that larval oyster *Crassostrea virginica* showed an acute sensitivity towards *Alexandrium catenella* and *Dinophysis acuminata* live cells (>50% inactivity and 21.9% mortality, respectively), but not to their purified toxins, saxitoxin and okadaic acid, respectively. The only purified toxin that shellfish larvae were sensitive to was pectenotoxin-2 from *D. acuminata* (PTX2; 49.6% mortality) [32]. It is well known that molluscs can accumulate phycotoxins due to their filter-feeding biology, making them toxin vectors and a risk to public health, which can cause the so-called shellfish poisonings, with a series of symptoms in humans according to the phycotoxin bioactivity, i.e., paralytic, diarrhetic, amnesic, and neurotoxic. A similar phenomenon has been observed in finfish, which can accumulate ciguatoxins that are causative of ciguatera fish poisoning in humans and that are produced by certain strains of benthic dinoflagellates of the genera *Gambierdiscus* and *Fukuyoa* that naturally occur in some tropical and sub-tropical coastal regions [2]. Bouquet et al. demonstrated that juvenile mullet finfish, *Liza ramada,* were able to accumulate pinnatoxins (PnTX) and portimines produced by the benthic dinoflagellate *Vulcanodinium rugosum*. Fish suffered no adverse effects, but cysts from this microalga were observed in faeces after fish were fed with live algal cells for three days. These cysts were able to germinate within 8–22 h after isolation from the faeces and incubation in an enriched seawater culture medium. Bouquet et al. recommended further investigations given that finfish may be vectors of pinnatoxins and may affect consumers, especially given that they observed pinnatoxin levels in fish tissues of up to 169.8 µg PnTX G kg^−1^, which were above the recommended sanitary level that so far has only been determined for shellfish (23 µg PnTX G kg^−1^) [33,34].

Palikova et al. observed that the cyanobacteria *Microcystis aeruginosa*, producer of microcystins, can worsen the health of the common carp *Cyprinus carpio* when infected with *Carp sprivivirus,* responsible for the Spring Viraemia of Carp viral disease (SVCV). During these exposure conditions, fish showed a weakened immune system and liver impairment compared with exposure to either *M. aeruginosa* or *C. sprivivirus* alone. This was concluded based on the increase in immunoglobulin production (up to ~15 mg mL^−1^ vs. <7 mg mL^−1^ in cyanobacteria plus SVCV exposures vs. controls or individual exposures, respectively), an increase in glucose (up to ~7 mmol L^−1^ vs. <5.6 mmol L^−1^ in the combined treatments vs. controls or SVCV exposures, respectively), and an increase in alanine aminotransferase activity, a marker for liver damage to confirm the hepatotoxicity by microcystins (up to 0.62 µkat L^−1^ vs. <0.47 µkat L^−1^ in combined exposures vs. controls or individual exposures, respectively) [35]. Rolton et al. contributed to this Special Issue with a review case study of New Zealand, which included an analysis of the current HAB situation and future views in preparation for the occurrence of new HAB events given the current presence of harmful species that have not formed blooms yet. It is suggested that due to the New Zealand Aquaculture Strategy for industry growth in the coming decades and the critical effect of climate change in the oceans, scientists, regulators, and seafood growers are feeling encouraged to start preparing for HAB scenarios that have otherwise been observed in other parts of the world with substantial economic losses. Of the three main farmed species in New Zealand, green-lipped mussels (*Perna canaliculus*) are at the top of production and revenue, followed by king (Chinook) salmon (*Oncorhynchus tshawytscha*) and Pacific oysters (*Crassostrea gigas*) [36]. Despite not having direct evidence of harmful algal species affecting farmed species, previous reports of salmon and green-lipped mussel mortalities and the decline in mussel spat settlement suggest the role of harmful phytoplankton given the frequent occurrence of HAB in growing areas, which appear to be expanding to other areas with a higher frequency and longer duration. Microalgal species that have so far formed HAB in New Zealand with sublethal or lethal effects in finfish and/or shellfish include *Pseudo-nitzschia australis*, *P. fraudulenta*, *P. multiseries*, *P. delicatissima*, *Pseudochattonella verruculosa*, *Alexandrium pacificum*, *A. minutum*, *Cerataulina pelagica*, *Gonyaulax fragilis*, *Dinophysis acuminata*, *Gymnodinium catenatum*, *Karenia brevisulcatum*, *K. mikimotoi*, *K. selliformis*, *K. umbrella*, *Ostreopsis* cf. *siamensis*, *Prymnesium calathiferum*, *Heterosigma akashiwo*, and *Fibrocapsa japonica*. Moreover, the species that pose a threat to New Zealand aquaculture industries due to their current presence despite not having formed blooms yet include *Alexandrium ostenfeldii*, *Margalefidinium polykrikoides*, *Heterocapsa* cf. *circularisquama*, *H. illdefina*, *Karlodinium veneficum*, *Pfiesteria piscicida*, *P. shumwayae*, *Prorocentrum rathymum*, *Chrysochromulina leadbeateri*, *Pavlomulina ranunculiformis*, *Chattonella antiqua*, and *C. marina* var. *antiqua*. Some of these species produce proper toxins that have been chemically described by other scientists, but other algal species are still being investigated to characterise their toxic compounds and specific bioactivity. The adverse effects that these algal species have on finfish and/or shellfish include mortality, filtration or swimming capacity reduction, larval development interruption, and growth rate reduction [37] (and other references therein).

Aquaculture is increasing globally, and its total production (including algae) has overtaken capture fisheries since 2013. Aquaculture production excluding algae (i.e., only aquatic animals) was 49% and capture fisheries production was 51% in 2020 worldwide; however, aquaculture has grown faster than capture fisheries in the last two years [38]. Therefore, it is crucial to continue monitoring harmful algal blooms since these events are one of the main threats to aquatic animals. Forecasting HAB and especially mitigating their impacts in the aquaculture industry still present challenges; however, it is critical to continue studying these events to create better management and mitigation programmes with a twofold aim: to minimise the economic losses while ensuring the welfare and survival of farmed species destined for human consumption.
